# Gastrointestinal and Variceal Bleeding Under Atezolizumab–Bevacizumab in Hepatocellular Carcinoma: Evidence from Trials to Real-World Practice

**DOI:** 10.3390/cancers18091432

**Published:** 2026-04-30

**Authors:** Hyo-Jin Lee, Hee Yeon Kim

**Affiliations:** 1 Department of Internal Medicine, Uijeongbu St. Mary’s Hospital, College of Medicine, The Catholic University of Korea, Seoul 06591, Republic of Korea; happyjinns@catholic.ac.kr; 2 Department of Internal Medicine, Bucheon St. Mary’s Hospital, College of Medicine, The Catholic University of Korea, Seoul 06591, Republic of Korea

**Keywords:** atezolizumab, bevacizumab, bleeding, hepatocellular carcinoma

## Abstract

Atezolizumab–bevacizumab is established as first-line therapy for unresectable hepatocellular carcinoma (HCC) based on phase III randomized evidence. Although overall safety outcomes were acceptable in the registration trial, the risk of gastrointestinal (GI) and variceal bleeding remains a clinically relevant concern, particularly in patients with cirrhosis and portal hypertension. Differences between trial-based safety estimates and observational data necessitate a focused evaluation of hemorrhagic risk in this setting. Randomized trial data indicate low rates of high-grade bleeding under protocol-driven endoscopic screening and predefined eligibility criteria. In contrast, real-world cohorts report higher incidences of GI and variceal hemorrhage, especially among patients with prior bleeding, untreated or high-risk varices, reduced hepatic reserve, and extensive portal vein tumor thrombosis. Pooled analyses confirm an increased prevalence of bleeding with atezolizumab–bevacizumab compared with non-antiangiogenic systemic therapies, although event rates vary across studies due to differences in patient selection and bleeding definitions. Severe and fatal hemorrhage occurs in a minority of cases and is concentrated in clinically high-risk subgroups. Bleeding during atezolizumab–bevacizumab therapy is influenced by baseline portal hypertension severity, hepatic functional status, and tumor-related vascular involvement. Trial-derived safety data reflect outcomes under controlled conditions and may underestimate risk in broader populations. Structured baseline assessment, endoscopic evaluation, and risk-adapted portal hypertension management are integral to clinical implementation. Prospective studies incorporating standardized hemorrhage definitions and predefined risk stratification frameworks are required to refine patient selection and optimize safety in routine practice.

## Simple Summary

Atezolizumab–bevacizumab has become a standard first-line therapy for advanced hepatocellular carcinoma (HCC). Although this regimen improves survival, it may increase the risk of gastrointestinal and variceal bleeding in patients with underlying cirrhosis and portal hypertension. Randomized trials reported relatively low rates of severe bleeding; however, observational studies from routine clinical practice suggest that bleeding events may occur more frequently, particularly in patients with impaired liver function, high-risk varices, or extensive vascular invasion. This review summarizes current evidence regarding the mechanisms, incidence, and clinical impact of bleeding during atezolizumab–bevacizumab therapy. It also discusses risk stratification and preventive strategies that may reduce hemorrhagic complications. A better understanding of bleeding risk is essential to optimize patient selection and improve the safety of this combination therapy in real-world settings.

## 1. Introduction

Hepatocellular carcinoma (HCC) remains a leading cause of cancer-related mortality worldwide, largely owing to its frequent diagnosis at an advanced stage and its close association with underlying cirrhosis and portal hypertension [1]. In recent years, systemic treatment options for advanced HCC have evolved substantially. The phase III IMbrave150 trial established the combination of atezolizumab, an anti-programmed death-ligand 1 (PD-L1) antibody, and bevacizumab, a vascular endothelial growth factor (VEGF) inhibitor, as the standard first-line therapy for unresectable HCC, demonstrating significant improvements in overall survival (OS) and progression-free survival (PFS) compared with sorafenib [2]. Consequently, atezolizumab–bevacizumab has been widely adopted in clinical practice. Despite its proven efficacy, the use of bevacizumab raises important safety concerns, particularly with respect to gastrointestinal (GI) and variceal bleeding [3]. Patients with HCC frequently have cirrhosis-related portal hypertension, esophageal or gastric varices, and impaired vascular integrity, all of which predispose them to bleeding complications [1,4]. VEGF inhibition may further exacerbate this vulnerability by impairing endothelial repair and vascular homeostasis [3,5].

In the IMbrave150 trial, the incidence of high-grade bleeding events was relatively low, and treatment-related fatal GI bleeding was uncommon [2]. Updated safety analyses with longer follow-up confirmed the overall favorable safety profile of the regimen but continued to report rare yet clinically meaningful fatal bleeding events [6,7]. In contrast, emerging real-world studies have reported a higher incidence of GI and variceal bleeding among patients treated with atezolizumab–bevacizumab. Prospective and retrospective observational cohorts have consistently shown that acute variceal bleeding occurs in a notable proportion of patients, often within the first few months of treatment [8,9,10]. These studies have identified prior variceal bleeding, large or high-risk varices, impaired liver function, and portal vein tumor thrombosis (PVTT) as major risk factors [7,9,10,11]. Furthermore, a recent systematic review and meta-analysis pooling data from multiple real-world cohorts demonstrated that the risk of variceal bleeding with atezolizumab–bevacizumab is not negligible and may be higher than that observed with tyrosine kinase inhibitors (TKIs) [12,13]. These findings highlight a discrepancy between randomized controlled trial (RCT) data and real-world experience regarding bleeding risk, suggesting that trial-based safety estimates may not fully capture the risk encountered in routine clinical practice.

Given the heterogeneity in study design, patient characteristics, and bleeding definitions, a comprehensive synthesis of the available evidence is required. The objective of this narrative review is to summarize and critically appraise the current evidence regarding GI and variceal bleeding risk following treatment with atezolizumab–bevacizumab in patients with HCC, with a focus on incidence, risk factors, clinical course, and implications for patient selection and management.

## 2. Pathophysiology

The bleeding risk observed during atezolizumab–bevacizumab therapy in HCC reflects the convergence of cirrhosis-related portal hypertension, tumor-driven vascular remodeling, and pharmacologic disruption of endothelial integrity. Because most patients with HCC have underlying cirrhosis, clinically significant portal hypertension and gastroesophageal varices are already present in a substantial proportion at treatment initiation, providing the anatomic substrate for hemorrhage [1,4].

At the molecular level, VEGF plays a central role in maintaining sinusoidal homeostasis through its regulation of endothelial nitric oxide synthase (eNOS) activity in liver sinusoidal endothelial cells (LSECs) [14]. Under physiological conditions, VEGF signaling via VEGF receptor 2 activates eNOS, sustaining the production of nitric oxide (NO) and the vasodilatory prostanoid prostacyclin [15]. Experimental evidence has demonstrated that targeted removal of VEGF signaling in transgenic mice results in loss of LSEC fenestration and the development of portal hypertension, with subsequent VEGF administration shown to ameliorate portal pressure [16]. Pharmacologic inhibition of VEGF signaling may disrupt endothelial NO-mediated vasodilation, potentially promoting intrahepatic vasoconstriction and increasing the portal pressure gradient [17,18], further amplifying hemorrhagic risk in the already-compromised cirrhotic vasculature.

Consistent with these molecular mechanisms, bevacizumab-mediated VEGF inhibition leads to regression of immature vessels, impaired endothelial repair, and increased microvascular fragility, thereby predisposing to mucosal and variceal bleeding [3]. Meta-analytic data across RCTs confirm that anti-VEGF monoclonal antibodies significantly increase both all-grade and high-grade bleeding risk, supporting a biologically plausible class effect that amplifies baseline hemorrhagic vulnerability in cirrhotic patients [5,19]. Beyond cirrhosis-related portal hypertension, extensive PVTT appears to further amplify hemorrhagic vulnerability by mechanically increasing portal pressure and destabilizing pre-existing collaterals. In a post hoc analysis of IMbrave150, patients with Vp4 portal vein invasion exhibited a markedly higher incidence of GI and fatal bleeding events, suggesting that tumor-driven hemodynamic distortion synergizes with VEGF inhibition to lower the bleeding threshold [7]. These findings highlight that bleeding risk is not solely determined by the presence of cirrhosis but is dynamically modulated by tumor burden and vascular invasion patterns.

**Figure 1:**
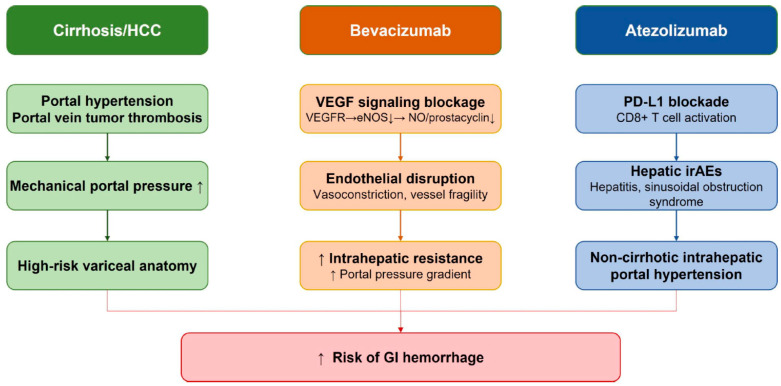
Mechanistic pathways leading to an increased gastrointestinal bleeding risk with atezolizumab–bevacizumab in hepatocellular carcinoma. HCC, hepatocellular carcinoma; VEGF, vascular endothelial growth factor; VEGFR, vascular endothelial growth factor receptor; eNOS, endothelial nitric oxide synthase; NO, nitric oxide; PD-L1, anti-programmed death-ligand 1; irAEs, immune-related adverse events.

The interaction between immune checkpoint inhibition and the vascular microenvironment is an area of active investigation. Preclinical data suggest that PD-L1 blockade may influence vascular endothelial signaling [20], though the clinical relevance of this interaction in patients with cirrhosis and portal hypertension has not been established. In addition, atezolizumab may independently destabilize portal hemodynamics through immune-related adverse events (irAEs) that directly target the hepatic microenvironment. Immune-mediated hepatitis and sinusoidal obstruction syndrome, irAEs associated with PD-L1 blockade, can cause focal or diffuse sinusoidal inflammation and obliteration, resulting in non-cirrhotic intrahepatic portal hypertension [21,22]. However, robust clinical data specifically quantifying the contribution of atezolizumab-mediated irAEs to portal pressure fluctuations in HCC remain limited (Figure 1).

## 3. Evidence from Studies

### 3.1. Evidence from RCTs

The pivotal evidence regarding bleeding risk under atezolizumab–bevacizumab derives primarily from the phase III IMbrave150 RCT, which established the combination as a first-line standard of care for unresectable HCC. In the primary analysis, atezolizumab–bevacizumab demonstrated a significant OS and PFS advantage over sorafenib, with a safety profile that was considered manageable and broadly consistent with the known toxicities of immune checkpoint inhibition and anti-VEGF therapy [2]. With respect to hemorrhagic complications, IMbrave150 reported relatively low rates of clinically significant bleeding. Upper GI bleeding and variceal hemorrhage were infrequent, and grade ≥ 3 bleeding events occurred in only a small proportion of patients, while fatal bleeding was rare [2]. These reassuring safety findings must be interpreted in the context of the trial’s strict eligibility criteria, which mandated baseline endoscopic screening and excluded or treated patients with high-risk varices prior to treatment initiation. This protocol-driven risk mitigation strategy likely contributed to the lower observed bleeding incidence compared with later real-world reports.

Extended follow-up analyses of IMbrave150 confirmed the durability of the safety profile over time. With longer observation, no late-emerging excess of hemorrhagic toxicity was identified, and the overall pattern of adverse events—including bleeding—remained stable, supporting the long-term tolerability of the regimen in carefully selected patients [6]. These findings indicate that, under optimized trial conditions, atezolizumab–bevacizumab does not appear to induce a cumulative or delayed bleeding risk. More granular insights into bleeding vulnerability have emerged from post hoc subgroup analyses. In patients with extensive PVTT (Vp4), a population characterized by more advanced disease and heightened portal hypertension, the incidence of GI bleeding was substantially higher than in the overall trial cohort. Notably, in this subgroup, any-grade GI bleeding approached 23%, grade ≥ 3 bleeding reached approximately 20%, and grade 5 GI hemorrhage—including esophageal variceal bleeding—was observed in a non-negligible fraction of patients [7]. Although the overall frequency of grade 3–4 treatment-related adverse events in this subgroup was paradoxically lower than that in the broader IMbrave150 population, the clustering of fatal bleeding events in Vp4 patients underscores that tumor-driven hemodynamic distortion can profoundly modify hemorrhagic risk even within an RCT framework.

Additional safety analyses have further nuanced the interpretation of RCT-derived bleeding risk. Exploratory studies suggest that hemorrhagic events may, at least in part, reflect on-target VEGF pathway inhibition and tumor vessel pruning rather than purely idiosyncratic toxicity, although this biological effect becomes maladaptive in the cirrhotic liver, where endothelial fragility and portal hypertension coexist [23]. From a clinical standpoint, these observations imply that even within an RCT context, bleeding risk is not uniform but is dynamically modulated by tumor burden, vascular invasion patterns, and baseline portal hemodynamics.

Overall, RCT data establish that atezolizumab–bevacizumab can be delivered with a relatively low incidence of major bleeding under controlled conditions. However, subgroup analyses reveal that patients with advanced vascular invasion represent a biologically distinct population with disproportionately elevated hemorrhagic risk. These RCT findings, therefore, provide both reassurance regarding overall regimen safety and an early signal that bleeding risk is highly context-dependent, consistent with the discrepancies later observed in real-world cohorts (Table 1).

### 3.2. Real-World Evidence of Bleeding Risk

Real-world studies have consistently demonstrated a higher incidence of GI and variceal bleeding under atezolizumab–bevacizumab than that reported in IMbrave150 [2], underscoring the limited external generalizability of RCT-derived safety estimates. In contrast to the highly selected trial population, real-world cohorts encompass a broader clinical spectrum, including patients with impaired liver function, untreated or high-risk varices, and extensive portal hypertension, all of which converge to amplify hemorrhagic vulnerability. A large real-world cohort analysis from a tertiary referral center reported a post-index GI bleeding rate of 17.2% across all systemic therapies, with bleeding risk reaching 23.4% in patients classified as high risk based on liver function, recent bleeding history, and variceal status. Within the subgroup treated specifically with atezolizumab–bevacizumab, the overall bleeding incidence was 17.3%, increasing to 19.4% in high-risk patients compared with only 5.9% in those deemed low risk [26]. These findings provide quantitative confirmation that the bleeding burden in routine practice substantially exceeds that observed in IMbrave150 [2]. Other real-world cohorts have reported concordant trends. Studies from Europe and East Asia consistently document variceal bleeding rates in the range of 7–14% under atezolizumab–bevacizumab, with prior variceal hemorrhage, high-risk varices, and deteriorated hepatic reserve emerging as dominant risk modifiers [9,10]. These cohorts also reveal that bleeding frequently necessitates transfusion, urgent endoscopic intervention, and temporary or permanent treatment discontinuation, highlighting the tangible clinical burden beyond mere event counts (Table 1).

However, real-world evidence is also characterized by substantial methodological heterogeneity. A systematic review and meta-analysis highlighted wide variability in bleeding definitions, inconsistent adverse-event reporting, and frequent under-ascertainment of severe complications, particularly in retrospective cohorts [12]. These limitations caution against overinterpreting pooled incidence estimates and underscore the need for standardized bleeding adjudication in future observational studies. To summarize, real-world data reveal that bleeding under atezolizumab–bevacizumab is both more frequent and more clinically consequential than suggested by RCTs, driven by broader patient inclusion, heterogeneous prophylactic strategies, and real-life deviations from trial protocols. These data support individualized risk stratification and proactive portal hypertension management when translating atezolizumab–bevacizumab from controlled trials into everyday clinical practice.

A critical interpretive caveat is that real-world bleeding estimates are substantially confounded by indication: clinicians preferentially assign atezolizumab–bevacizumab to patients with more advanced disease, impaired hepatic reserve, or macrovascular invasion—the population at greatest intrinsic hemorrhagic risk. This systematic enrichment of high-risk individuals means that observed bleeding rates reflect, at least in part, the underlying severity of the treated population rather than drug-attributable toxicity. Accordingly, real-world bleeding incidence should be interpreted as an upper-bound estimate, and direct numerical comparisons with RCT-derived rates must be approached with caution.

### 3.3. RCT vs. Real-World Bleeding Risk: Comparative Interpretation

A consistent and clinically salient discrepancy emerges when comparing bleeding risk under atezolizumab–bevacizumab between RCTs and real-world studies. This discrepancy largely reflects differences in patient selection and trial protocol. IMbrave150 enforced mandatory baseline endoscopic screening and excluded or pre-treated patients with high-risk varices, thereby systematically depleting the trial population of those at greatest hemorrhagic risk [2]. In contrast, real-world cohorts encompass broader clinical spectra, including patients with untreated or high-risk varices, impaired liver function, and extensive portal hypertension [9,10].

Differences in outcome definition and ascertainment further magnify this apparent gap. RCTs rely on standardized Common Terminology Criteria for Adverse Events (CTCAE) grading and prospectively adjudicated adverse events, whereas real-world studies often capture “clinically significant bleeding” based on transfusion requirements, endoscopic intervention, or hospitalization. This definitional asymmetry inflates real-world incidence estimates relative to trial figures, while simultaneously underestimating severe bleeding in observational datasets due to incomplete adverse-event reporting [12]. Thus, RCTs may underrepresent the true bleeding burden by design, whereas real-world studies may overrepresent it through broader clinical capture but suffer from inconsistent documentation.

Subgroup analyses within IMbrave150 provide an internal bridge between these two evidence domains. In patients with extensive PVTT, GI bleeding rates rose dramatically, and fatal hemorrhagic events clustered within this high-risk population [7]. These findings demonstrate that, even under RCT conditions, bleeding risk is highly context-dependent and that trial-averaged safety metrics obscure clinically decisive heterogeneity.

## 4. Risk Factors for Bleeding Under Atezolizumab–Bevacizumab

Bleeding events under atezolizumab–bevacizumab vary according to portal hypertension severity, hepatic functional reserve, and tumor burden. Among portal hypertension-related factors, the presence of high-risk gastroesophageal varices and a prior history of variceal bleeding emerge as the most robust predictors of subsequent hemorrhage. In a real-world cohort study, patients with a prior history of variceal bleeding had a substantially higher risk of recurrent hemorrhage during atezolizumab–bevacizumab therapy, indicating that previous bleeding is a strong predictor of subsequent events in the setting of portal hypertension [9]. Similarly, Park et al. reported that untreated or high-risk varices independently predicted variceal bleeding, even after adjustment for other clinical covariates [10]. These observations are mechanistically consistent with portal hypertension physiology, in which elevated wall tension and collateral vessel fragility lower the hemorrhagic threshold when anti-VEGF therapy is introduced.

Liver functional reserve constitutes a second major risk axis. Meta-analyses focusing on patients with impaired hepatic function have shown that those with Child–Pugh class B or higher albumin–bilirubin (ALBI) grades experience significantly higher rates of grade ≥ 3 adverse events, including bleeding [11]. In a bleeding-focused meta-analysis, meta-regression identified a higher proportion of ALBI grade 3 patients as an independent risk factor for hemorrhagic events, highlighting that subtle differences in hepatic reserve within Child–Pugh A populations also translate into clinically meaningful risk gradients [13].

Tumor-related vascular features further modulate bleeding susceptibility. Extensive PVTT has been associated with a disproportionately high incidence of GI and fatal bleeding in post hoc analyses of IMbrave150, reflecting the additive effects of mechanical portal flow obstruction and collateral destabilization [7]. Beyond intravascular invasion, recent real-world data suggest that extraluminal varices detected on cross-sectional imaging carry similar hemorrhagic implications to endoscopically visible varices, reinforcing the concept that structural vascular abnormalities—whether luminal or extraluminal—are clinically meaningful bleeding substrates [27].

Patient-related factors also contribute to risk heterogeneity. A recent meta-analysis identified a higher body mass index as an independent predictor of bleeding, potentially reflecting more advanced portal hypertension or distinct metabolic liver disease phenotypes with heightened vascular vulnerability [13]. Finally, drug-related class effects must be acknowledged. Bevacizumab, as an anti-VEGF monoclonal antibody, is intrinsically associated with an increased risk of bleeding across tumor types. Large meta-analyses of randomized trials confirm significantly elevated risks of both all-grade and high-grade hemorrhage with bevacizumab, providing a mechanistic backdrop against which portal hypertension-related vulnerabilities are amplified in cirrhotic patients [19].

## 5. Pooled Evidence from Systematic Reviews and Meta-Analyses

Systematic reviews and meta-analyses provide an important quantitative framework for interpreting the bleeding signal observed across both randomized and real-world datasets. While individual studies vary in design and bleeding definitions, pooled analyses consistently confirm that hemorrhagic events under atezolizumab–bevacizumab are uncommon overall but clinically consequential when they occur, with GI—predominantly variceal—bleeding representing the dominant phenotype [13,24]. In a recent bleeding-focused meta-analysis encompassing 28 studies and nearly 4000 patients, the combination of atezolizumab–bevacizumab was associated with a significantly higher prevalence of bleeding compared with TKIs, supporting the biological premise that VEGF inhibition may exacerbate vascular fragility in the cirrhotic liver [13]. A comprehensive systematic review and meta-analysis of 47 real-world studies confirmed that overall adverse event rates under atezolizumab–bevacizumab were broadly comparable to those observed with TKIs, yet highlighted persistent concerns regarding variceal bleeding, particularly in patients with advanced cirrhosis and portal hypertension [24]. These findings support the notion that bleeding is not an idiosyncratic outlier event but a reproducible safety signal across diverse clinical settings.

Meta-analyses focusing on special populations further delineate risk gradients. In patients with impaired liver function, pooled data indicate significantly higher rates of grade ≥ 3 adverse events—including bleeding—among those with Child–Pugh class B or higher ALBI grades [11]. Complementing this observation, meta-regression in the bleeding-focused meta-analysis identified a higher proportion of ALBI grade 3 patients as an independent predictor of hemorrhagic events, underscoring that subtle decrements in hepatic reserve materially translate into elevated bleeding risk [13].

However, pooled estimates must be interpreted with caution due to pronounced methodological heterogeneity. A systematic review examining adverse-event reporting practices revealed wide variability in bleeding definitions, inconsistent grading thresholds, and frequent under-ascertainment of severe complications—particularly in retrospective real-world cohorts [12]. These limitations introduce uncertainty into pooled incidence figures and likely contribute to both underestimation of fatal bleeding in observational studies and inflation of apparent bleeding rates when broader “clinically significant bleeding” definitions are applied.

Network meta-analyses comparing first-line systemic regimens provide an additional interpretive layer. While atezolizumab–bevacizumab consistently ranks among the most effective therapies in terms of OS, it also exhibits a higher probability of severe adverse events compared with immune checkpoint inhibitor monotherapies, reflecting an inherent efficacy–toxicity trade-off [28,29]. Although these analyses do not isolate bleeding as a standalone endpoint, their tolerability rankings align with the bleeding-focused meta-analytic signal that atezolizumab–bevacizumab carries a distinct hemorrhagic burden.

## 6. Timing, Severity, and Clinical Course of Bleeding Events

Bleeding events associated with atezolizumab–bevacizumab in HCC follow characteristic temporal patterns, display a broad severity spectrum, and exert clinically meaningful downstream consequences. In the primary analysis of IMbrave150, hemorrhagic events were relatively infrequent, with upper GI bleeding and variceal hemorrhage constituting the dominant phenotypes [2]. Most bleeding events occurred early following treatment initiation, consistent with the known early effects of VEGF inhibition on vascular integrity and the destabilization of pre-existing portal hypertensive collaterals. Extended follow-up confirmed that the overall bleeding profile remained stable over time, without an emergent late excess of hemorrhagic toxicity [6]. These findings suggest that atezolizumab–bevacizumab does not induce a cumulative or delayed bleeding risk under optimized trial conditions. More granular insights into timing and severity have emerged from post hoc subgroup analyses. In patients with extensive PVTT, GI bleeding clustered disproportionately early and was frequently severe [7]. These fatal bleeding events were largely confined to Vp4 patients, underscoring that tumor-driven portal hemodynamic distortion accelerates both the onset and clinical impact of hemorrhagic complications.

Pooled evidence from systematic reviews and meta-analyses further clarifies the severity distribution of bleeding events. A bleeding-focused meta-analysis reported a pooled prevalence of grade ≥ 3 bleeding of 4.42% and fatal bleeding of 2.06%, indicating that while most bleeding events are manageable, a clinically significant minority progress to life-threatening hemorrhage [13]. These pooled estimates contextualize the IMbrave150 findings by demonstrating that severe and fatal bleeding are not merely idiosyncratic anomalies but reproducible outcomes across diverse clinical settings. Real-world cohorts provide additional insight into the clinical course of bleeding. Observational studies consistently show that bleeding events frequently necessitate transfusion, urgent endoscopic intervention, and temporary or permanent treatment interruption [9,10]. In a large real-world cohort, post-index GI bleeding occurred in 17.2% of patients, with substantially higher rates in those classified as high risk [26]. These findings underscore that, outside trial conditions, bleeding is not only more frequent but also more clinically disruptive.

The prognostic implications of bleeding appear more nuanced than a simple toxicity–outcome dichotomy. Propensity score-adjusted analyses indicate that the occurrence of variceal bleeding does not necessarily translate into inferior OS when bleeding is promptly recognized and appropriately managed, suggesting that hemorrhagic events, while clinically destabilizing, do not uniformly negate the oncologic benefit of atezolizumab–bevacizumab [27]. These findings have practical implications for treatment continuation decisions, supporting the feasibility of continuing therapy in selected patients after stabilization.

In aggregate, these data delineate a bleeding risk profile characterized by early onset, a wide severity spectrum, and clinically meaningful downstream consequences. While most bleeding events are manageable with timely intervention, a non-trivial proportion progress to severe or fatal hemorrhage, particularly in patients with advanced portal vein invasion and severe portal hypertension. These insights underscore the importance of early vigilance, proactive risk stratification, and individualized management strategies when implementing atezolizumab–bevacizumab therapy in routine clinical practice.

## 7. Risk Mitigation Strategies

Bleeding prevention must be integrated into systemic therapy planning rather than managed reactively after event onset.

### 7.1. Baseline Risk Stratification Before Atezolizumab–Bevacizumab Initiation

**Figure 2:**
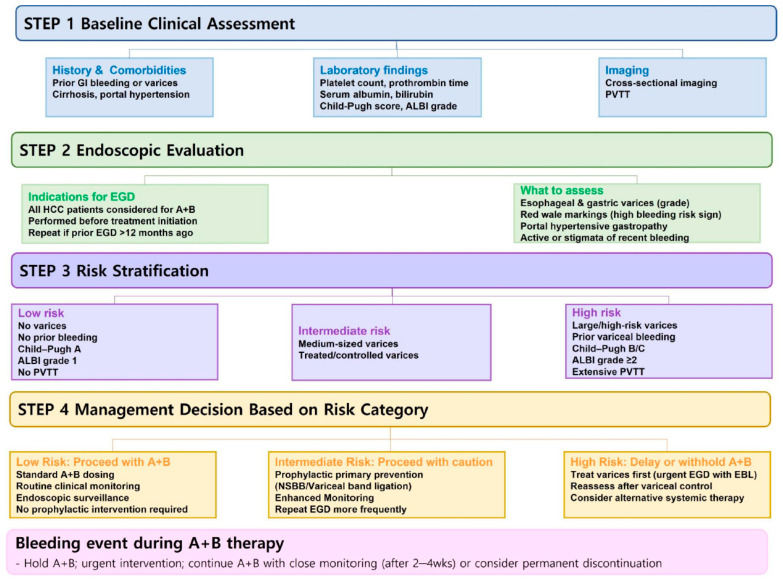
Suggested clinical approach to bleeding risk assessment before and during atezolizumab–bevacizumab therapy. ALBI grade, albumin–bilirubin grade; PVTT, portal vein tumor thrombosis; HCC, hepatocellular carcinoma; A + B, atezolizumab + bevacizumab; EGD, esophagogastroduodenoscopy; NSBB, non-selective beta-blocker; EBL, endoscopic band ligation.

A pragmatic and evidence-informed approach is to stratify bleeding risk along three principal axes: (i) portal hypertension severity, (ii) hepatic functional reserve, and (iii) tumor burden. Patients with high-risk gastroesophageal varices, prior variceal bleeding, Child–Pugh class B status, ALBI grade ≥ 2, or PVTT consistently exhibit an increased hemorrhagic risk (Table 2) [9,10,11]. In a US real-world retrospective cohort, patients were categorized as having ‘GI bleeding risk’ if they met pre-specified criteria (Child–Pugh B/C, pre-index GI bleeding, uncontrolled hypertension, or significant varices with band ligation). Post-index GI bleeding with atezolizumab–bevacizumab occurred in 19.4% of the risk group versus 5.9% of the non-risk group [26]. These findings support the routine incorporation of formal bleeding risk stratification into treatment decision-making (Figure 2).

### 7.2. Endoscopy Timing and Surveillance Strategy

Hepatology–oncology guidance documents commonly recommend upper endoscopy prior to initiating atezolizumab–bevacizumab, followed by periodic reassessment during therapy [4,8]. Because HCC itself can confound non-invasive portal hypertension markers, expert reviews continue to emphasize the central role of endoscopic evaluation even in patients who might otherwise meet favorable non-invasive criteria [30]. Recent real-world data further suggest that extraluminal varices detected on cross-sectional imaging carry similar hemorrhagic implications to intraluminal varices, reinforcing that vascular vulnerability may be present even when endoscopy has not yet been performed [27].

### 7.3. Primary Prophylaxis: Non-Selective Beta-Blockers First, with Selective Use of Band Ligation

For primary prophylaxis of variceal bleeding in HCC, expert opinion and consensus statements favor non-selective beta-blockers (NSBBs) as first-line therapy, given their systemic portal pressure-lowering effects and lack of mucosal injury risk [4,30]. Endoscopic band ligation (EBL) remains appropriate in patients with contraindications or intolerance to NSBBs, but its timing should be carefully coordinated with anti-VEGF exposure, as mucosal ulceration following EBL may transiently increase bleeding susceptibility [8]. These considerations argue against routine, reflexive EBL in all patients and instead support individualized prophylactic strategies. The optimal timing of bevacizumab interruption and resumption around endoscopic variceal interventions represents one of the most clinically relevant yet unresolved issues in the management of patients receiving atezolizumab–bevacizumab therapy. Although there are no prospective data specifically defining optimal timing around endoscopic variceal therapy, the following recommendations are derived from bevacizumab pharmacokinetics, general oncologic practice, and available expert opinion: Bevacizumab has a mean plasma half-life of approximately 20 days (range 11–50 days), and its anti-VEGF activity impairs normal wound healing by inhibiting angiogenesis and vascular repair [31,32]. In general oncologic practice, it is recommended to withhold bevacizumab for at least 4–6 weeks before elective surgery and to delay resumption for at least 28 days postoperatively, or until the surgical wound is fully healed [32,33]. For less invasive procedures, the duration of bevacizumab interruption can be reduced to 7 to 14 days [34]. Although EBL is far less invasive than surgery, it consistently produces post-banding mucosal ulcers that typically heal within 2–3 weeks in non-anticoagulated, non-bevacizumab-treated patients but may be substantially delayed under ongoing anti-VEGF therapy [35]. Based on the bevacizumab wound-healing data and the known risk of post-banding ulcer bleeding peaking within the first 10–14 days, we recommend a minimum interval of 2 weeks between the last EBL session and the first dose of atezolizumab–bevacizumab. An expert opinion similarly recommends that atezolizumab–bevacizumab should not be initiated within 14 days after ligation, as the bleeding risk is highest within the first 10 days, and advises repeat endoscopy to re-evaluate the status of varices and bands. Importantly, in expert opinion, atezolizumab–bevacizumab treatment should not be delayed until complete variceal eradication, as eradication requires 3–4 EBL sessions at 2–3 week intervals [36].

### 7.4. Acute Variceal Bleeding During Treatment

When acute variceal hemorrhage occurs during atezolizumab–bevacizumab therapy, both drugs should be immediately held. Management should follow established principles of portal hypertension, with emphasis on portal pressure reduction and prompt hemostatic control [4]. Available real-world data indicate that, with timely and appropriate intervention, bleeding events do not necessarily require permanent discontinuation of atezolizumab–bevacizumab and may not adversely affect OS [27]. Decisions regarding resumption of bevacizumab after acute variceal hemorrhage should be individualized and guided by multidisciplinary assessment, taking into account the effectiveness of variceal control and the patient’s clinical stability [36,37]. In this context, continuation of atezolizumab monotherapy during the bevacizumab-free interval has been proposed based on expert opinion, although supporting evidence remains limited [36]. Whether atezolizumab–bevacizumab should be permanently discontinued after acute variceal bleeding or whether alternative anti-angiogenic agents such as tyrosine kinase inhibitors provide a safer option remains uncertain and requires prospective validation.

### 7.5. Selecting Alternative Regimens in High-Risk Patients

In clinical practice, the bleeding risk associated with atezolizumab–bevacizumab should be interpreted in the context of alternative systemic therapies. Although direct head-to-head comparisons remain limited, available evidence suggests that anti-VEGF-based regimens may confer a higher risk of GI and variceal bleeding than non-VEGF-targeting treatments, including TKIs [13]. In addition, the STRIDE regimen (durvalumab–tremelimumab) demonstrated a significantly lower risk of treatment-emergent bleeding events, with grade 3/4 bleeding occurring in only 0.5% of patients [38]. Therefore, for patients judged to be at prohibitive bleeding risk—such as those with refractory high-risk varices, recurrent recent variceal hemorrhage, or severely impaired hepatic reserve—expert reviews increasingly discuss immune checkpoint inhibitor-only regimens as a potential alternative to anti-VEGF-based combinations while acknowledging the current lack of prospective comparative evidence [8]. Although such strategies may modestly compromise antitumor efficacy, they may offer a more favorable safety trade-off in carefully selected individuals.

## 8. Limitations

This narrative review has several important limitations that warrant careful consideration. First, the literature selection process was inherently non-systematic and subject to narrative review bias. Although major randomized trials, large real-world cohorts, and key meta-analyses were prioritized, the possibility of incomplete study capture and selective emphasis cannot be excluded.

Second, substantial heterogeneity exists across the included studies in terms of patient populations, bleeding definitions, and outcome ascertainment. RCTs relied on standardized CTCAE grading, whereas real-world studies often defined bleeding based on transfusion requirements, hospitalization, or endoscopic intervention. This definitional asymmetry complicates direct comparisons and may inflate real-world incidence estimates while simultaneously under-ascertaining severe events in retrospective cohorts.

Third, confounding by indication represents a critical limitation of observational data. In routine practice, clinicians may preferentially prescribe atezolizumab–bevacizumab to patients with more advanced disease or limited therapeutic alternatives, inadvertently enriching real-world cohorts with individuals at intrinsically higher hemorrhagic risk. This dynamic likely contributes to the observed discrepancy between trial-based and real-world bleeding rates.

Fourth, heterogeneity in portal hypertension prophylaxis strategies—including baseline endoscopy utilization, NSBB prescription, timing of endoscopic band ligation, and bevacizumab interruption practices—introduces additional inter-study variability that cannot be adequately adjusted for in pooled analyses.

Fifth, bleeding risk is not static over time, and few studies incorporated time-varying risk models or competing risk frameworks. Early-onset hemorrhage, variceal progression during therapy, and mortality-related censoring may therefore be underappreciated.

Finally, the evidence base remains imbalanced, with one pivotal randomized trial underpinning efficacy and safety estimates, contrasted with a predominance of retrospective real-world studies and heterogeneous meta-analyses. Prospective studies incorporating standardized bleeding definitions, protocolized prophylaxis, and risk-adapted treatment algorithms are urgently needed to refine individualized bleeding risk prediction under atezolizumab–bevacizumab therapy.

## 9. Conclusions

Atezolizumab–bevacizumab has transformed first-line therapy for advanced HCC, yet its survival benefit is accompanied by a clinically meaningful, context-dependent risk of GI bleeding that demands systematic appraisal. RCTs likely underestimate the true bleeding burden due to stringent eligibility criteria, including the exclusion of patients with untreated or high-risk varices. In contrast, real-world evidence reveals substantially higher bleeding rates, particularly in patients with portal hypertension, impaired liver function, and extensive vascular invasion. This discordance is further compounded by confounding by indication, wherein patients with the most advanced disease are disproportionately represented in observational datasets. These findings underscore the importance of integrating bleeding risk assessment into routine clinical decision-making. Individualized risk stratification, systematic pre-treatment endoscopic evaluation, and proactive portal hypertension management should be considered essential components of care. In addition, careful monitoring during the early treatment period and a multidisciplinary approach to the management of bleeding events are critical to maintaining treatment continuity and maximizing therapeutic benefit. Despite growing evidence, several uncertainties remain, including the optimal management of patients at high bleeding risk, the timing of anti-VEGF therapy around endoscopic interventions, and the role of alternative systemic therapies in this setting. Future prospective studies are needed to refine risk-adapted strategies and to better define the balance between efficacy and safety. Ultimately, a more nuanced, patient-centered approach will be required to ensure that the survival benefits of atezolizumab–bevacizumab are translated into durable real-world outcomes.

## Figures and Tables

**Table 1:** Key studies evaluating bleeding risk under atezolizumab–bevacizumab in hepatocellular carcinoma.

Study (First Author, Year)	Study Design	Population (n)	Baseline Portal HTN/Varices	Bleeding Definition	Bleeding Incidence	Key Findings
Finn, 2020 [2]	Phase III RCT	501	Mandatory EGD; high-risk varices excluded	CTCAE	Upper GI bleeding ~3–4%	Low bleeding under strict selection
Cheng, 2022 [6]	RCT follow-up	501	Same as IMbrave150	CTCAE	Stable over time	No late bleeding signal
Larrey, 2022 [9]	Real-world cohort	43	Mixed; prior bleeding included	Clinical bleeding	Variceal~14%	Prior bleeding predicts recurrence
Park, 2025 [10]	Real-world cohort	640	Mixed	Clinical bleeding	Variceal~7%	A low platelet count, main PVI, history of GI bleeding, and varices needing treatment were significant risk factors
Song, 2024 [13]	SR & meta-analysis	3895	Mixed	Any/grade ≥ 3/fatal	Any 8.4%; grade ≥ 3 4.4%; GI bleeding 5.5%	Higher vs. TKIs
Kulkarni, 2023 [24]	SR & meta-analysis	5400	Mixed	Any-grade AE	Variceal~4.7%	Higher incidence of variceal bleeding in patients with main PVT
Manfredi, 2025 [25]	Real-world meta-analysis	2179	Mixed	Clinical bleeding	Variceal~9%	Rates of GI bleeding were higher compared with prospective clinical trials

**Table 2:** Risk stratification for gastrointestinal bleeding in patients receiving atezolizumab–bevacizumab.

Risk Level	Clinical Features
Low	No varices; No prior bleeding; Child–Pugh A; ALBI grade 1; No PVTT
Medium	Medium-sized varices; Treated/controlled varices
High	Large/high-risk varices; Prior variceal bleeding; Child–Pugh B/C; ALBI grade ≥ 2; Extensive PVTT

ALBI grade, albumin–bilirubin grade; PVTT, portal vein tumor thrombosis.

## Data Availability

No new data were created or analyzed in this study. Data sharing is not applicable.

## Abbreviations

The following abbreviations are used in this manuscript:

HCC	hepatocellular carcinoma
PD-L1	anti-programmed death-ligand 1
VEGF	vascular endothelial growth factor
OS	overall survival
PFS	progression-free survival
GI	gastrointestinal
PVTT	portal vein tumor thrombosis
TKI	tyrosine kinase inhibitor
RCT	randomized controlled trial
eNOS	endothelial nitric oxide synthase
LSECs	liver sinusoidal endothelial cells
NO	nitric oxide
IrAE	immune-related adverse events
CTCAE	Common Terminology Criteria for Adverse Events
ALBI	Albumin–bilirubin
NSBB	non-selective beta-blocker
EBL	endoscopic band ligation
